# Effects of 12-week pilates reformer training on the biomechanics of Latin dance Cha-Cha circle chasing technique

**DOI:** 10.3389/fphys.2025.1549389

**Published:** 2025-06-04

**Authors:** Li Che, Yijia Zhou, Ying Wang

**Affiliations:** ^1^ School of Dance and Martial Arts, Capital University of Physical Education and Sports, Beijing, China; ^2^ Institute of Physical Education and Training, Capital University of Physical Education and Sports, Beijing, China

**Keywords:** pilates reformer training, biomechanics, Cha-Cha circle chasing, joint angle, muscle force

## Abstract

**Background:**

Pilates Reformer training is a scientifically validated method for enhancing body control. However, research on its application to improve the technical quality of Latin dance movements, particularly the Cha-Cha circular chase step, is limited. This study aims to evaluate the effectiveness of Pilates Reformer training in enhancing this technique, with the goal of providing a theoretical foundation for its use in improving Latin dance performance and preventing injuries.

**Methods:**

Our study involved 18 Latin dance students specializing in sports dance, aged 18–30 years, with at least 3 years of Latin dance experience. Participants were randomly assigned to an experimental group (n = 9) and a control group (n = 9). Over a 12-week period, the experimental group underwent Pilates Reformer training, while the control group continued with their regular training. Biomechanical data were collected before and after each session to assess joint angles, movement speed, muscle activation, and technical quality scores using the WDSF 3.0 evaluation criteria. Data analysis and visualization were performed using Graph Prism 10.0 software, and statistical analyses were conducted with SPSS software (Version 22). Paired-sample t-tests were used for within-group comparisons, and independent-sample t-tests were used for between-group comparisons, with a significance level set at p < 0.05.

**Results:**

(1) The experimental group showed significant (P < 0.05) or highly significant (P < 0.01) differences in the joint angles of the hip, knee, and ankle at key points across all three axes (X, Y, Z) compared to the control group. The experimental group also exhibited significant improvements (P < 0.05) in the flexion and extension speeds of these joints during the movement phases, while the control group showed minimal changes (P > 0.05). (2) Pilates Reformer training led to significant increases (P < 0.05 or P < 0.01) in the RMS normalized muscle activation values in both legs during the Cha-Cha circular chase step. In contrast, the control group showed increased iEMG values, but these did not reach statistical significance (P > 0.05). (3) The experimental group showed significant improvements (P < 0.05) in posture (B1), foot movements (B4), and Latin characteristics (B5), as well as highly significant improvements (P < 0.01) in basic movements (B6) and preparation-action-recovery (B7) compared to the control group. Additionally, the experimental group exhibited consistent improvements in the average TQ scores across all evaluated components.

**Conclusion:**

Pilates Reformer training significantly enhances joint angles, movement speeds, muscle activation, and technical quality in Latin dance, particularly the Cha-Cha circular chase step. These findings demonstrate the potential of Pilates Reformer training to improve dance technique, optimize movement quality, and support injury prevention in Latin dance athletes, providing strong evidence for its effectiveness in this domain.

## 1 Introduction

With the rising popularity of Latin dance, the community of dance enthusiasts continues to expand, underscoring the importance of providing scientifically grounded guidance. For Latin dancers, mastering technical skills through consistent practice and professional training is essential. Latin dance encompasses various styles, each characterized by unique features such as movement patterns in basic techniques, methods of center-of-gravity transfer, and the integration of body movements with rhythm (Krasnow et al., 2011). Cha-Cha, known for its passionate, dynamic, and energetic style, emerged as a prominent Latin dance in the 1950s and was later standardized by the Imperial Society of Teachers of Dancing (ISTD) with defined steps and dance rules. Cha-Cha is recognized for its fast-paced, powerful, and intricately balanced technical combinations, as well as its ability to showcase individual expression. High technical scores in Cha-Cha are often attributed to solid foundational training and the execution of high-quality technical movements (Wilson and Kwon, 2008).

Latin dance is a complex, full-body activity that requires high levels of physical engagement. Research has shown a positive correlation between dancers’ range of motion and speed with the audience’s aesthetic experience (Neave et al., 2010; Torrents et al., 2013; McCarty et al., 2013; Chang et al., 2016), indicating that skilled dancers excel in these aspects. Moreover, these variables likely reflect a dancer’s motor skills and coordination (Chang et al., 2016). From a biomechanical perspective, Latin dance places significant physical demands on dancers, particularly in lower limb control, muscle strength, balance, flexibility, and coordination (Liu et al., 2022; Kiliç and Nalbant, 2022). Studies comparing kinematic variables of Cha-Cha-Cha technical movements among dancers of different skill levels (beginner, intermediate, expert) have revealed that joint velocity and range of motion significantly increase with skill level (Chang et al., 2020). Despite these findings, there is relatively little research on the biomechanics of the lower limbs in Latin dance compared to other sports. Alarmingly, recent studies have highlighted the prevalence of severe lower limb injuries among dancers during training (Kuisis et al., 2012). This underscores the critical role of the lower limbs in dance performance, where any injury could have profound implications for a dancer’s career. Therefore, in-depth research into the biomechanical characteristics of dancers’ lower limbs is of paramount importance.

Pilates was developed by Joseph Pilates in the 1920s (Latey, 2001). It is an exercise method that integrates full-body movement, breathing, focus, precision, and rhythm (Latey, 2002), characterized by its emphasis on accuracy and control (Kibler et al., 2006). Pilates encompasses a variety of exercise forms, including aerobic, resistance, and strength training, and can be performed using specialized equipment (equipment-based Pilates) or on a mat without equipment (mat-based Pilates) (Kamioka et al., 2016). The Reformer is a resistance training apparatus designed by Joseph Pilates. It consists of a platform that moves along tracks, with resistance provided by the user’s body weight and springs attached to the tracks and platform. This training adheres to the principle of overload. Reformer training has been shown to enhance muscle strength, flexibility, and core stability, as well as improve control of the trunk and pelvis, posture, and breathing efficiency (Wells et al., 2012; Kliziene et al., 2017). Furthermore, research has demonstrated the positive effects of Pilates on various health parameters (Granacher et al., 2013; de Oliveira et al., 2015; Mazzarino et al., 2015). Pilates not only improves flexibility, balance, posture alignment, and functional capacity but also optimizes body composition and reduces the risk of falls (Cruz Ferreira et al., 2011; Engers et al., 2016).

In recent years, Pilates has achieved remarkable advancements across various fields and has been widely applied to enhance performance in competitive sports such as football (Parolini et al., 2024), basketball (Chouhan et al., 2022), and volleyball (Jinglong, 2023), as well as to improve functionality in the field of sports rehabilitation (Patti et al., 2021; Göz et al., 2023; Tarnas et al., 2024). These applications have garnered widespread recognition. However, research focusing on the biomechanics of Cha-Cha technical movements remains limited, and there is a notable lack of studies investigating the use of Pilates Reformer training as part of Latin dancers’ exercise programs to improve technical movement quality. This study aims to explore the effects of a 12-week Pilates Reformer training program on the kinematic parameters of Cha-Cha circular chase steps and lower limb muscle strength. We anticipate that the preliminary findings of this study will provide valuable insights for designing future research and developing Pilates-based training programs for Latin dancers.

## 2 Participants and methods

### 2.1 Participants

This study recruited 18 Latin dance students specializing in sports dance from the Capital University of Physical Education and Sports. Among them, 10 were female students with an average age of 21.00 ± 1.94 years, an average height of 165.30 ± 3.74 cm, and an average weight of 51.15 ± 3.37 kg. The remaining eight participants were male students with an average age of 20.75 ± 2.05 years, an average height of 179.50 ± 4.28 cm, and an average weight of 71.20 ± 4.66 kg (see Table 1 for details).

**TABLE 1:** Demographic characteristics of participants.

Group	Participant	Sex	Age (year)	Weight (kg)	Height (cm)	BMI (kg/m^2^)
Experimental group	1	Female	19	50	167	17.93
	2	Female	24	43	156	17.67
	3	Female	21	55	166	19.96
	4	Female	19	51	164	18.96
	5	Female	19	51	164	18.96
	6	Male	23	65	172	21.97
	7	Male	19	67	175	21.88
	8	Male	21	72.5	178	22.88
	9	Male	22	77	185	22.50
Control group	1	Female	24	54	170	18.69
	2	Male	24	73.1	183	21.83
	3	Female	22	54	165	19.83
	4	Female	20	51	166	18.51
	5	Male	19	66	181	20.15
	6	Female	20	52.5	168	18.60
	7	Male	19	76.5	181	23.35
	8	Male	19	72.5	181	22.13
	9	Female	22	50	167	17.93

The inclusion criteria for participant recruitment were as follows:

(1) Participants were healthy young Latin dance students enrolled in the sports dance program at the Capital University of Physical Education and Sports.
(2) Participants had at least 3 years of Latin dance training experience.
(3) Participants engaged in Latin dance training at least three times per week, with each session lasting no less than 90 min.
(4) Participants had not experienced any injuries in the past 6 months.
(5) Participants had no prior experience with Pilates Reformer training.

This study recruited 18 Latin dance students specializing in sports dance from the Capital University of Physical Education and Sports. Prior to data collection, all participants were thoroughly informed about the study’s purpose, procedures, conditions, and requirements. Detailed information about the study was provided in a written consent form, which was signed by each participant to confirm their understanding and agreement. The study was conducted in accordance with the Declaration of Helsinki and approved by the ethics Committee of Capital University of Physical Education and Sports (Protocol Code: 2024A194).

### 2.2 Procedures

A 12-week training intervention was implemented in this study. The first 2 weeks were dedicated to educating and guiding participants, helping them familiarize themselves with the intervention process and exercise content. It was explicitly stated that during the training period, participants were prohibited from engaging in any additional Pilates Reformer training or other supplementary specialized training outside the classroom exercises, ensuring the effectiveness of the intervention. Both groups completed three training sessions per week, scheduled on Tuesday, Thursday, and Saturday afternoons from 1:30 p.m. to 3:00 p.m., with each session lasting 90 min. The Pilates Reformer training was conducted by a certified Pilates instructor, while the conventional technical training was guided by a Latin dance coach based on their prior training experience.

Both training programs in this study were divided into three phases: (a) Warm-up Phase: A 10-min session of warm-up exercises. (b) Main Training Phase: A 60–65-min session focused on primary training activities. (c) Stretching Phase: A 15-min session of stretching exercises. All participants were in their off-season during the study period and had no competition schedules.

During the study planning phase, five experts with experience in sports dance training and four professional Pilates Reformer instructors jointly evaluated the content validity of the training intervention and the methods for outcome assessment. According to Lynn (1986), there is no universally accepted standard for the number of experts in content validity evaluations, with recommendations typically ranging from 3 to more than 10 experts. In this study, the content validity of the intervention and the evaluation tools were assessed by 10 field experts through a questionnaire survey. The questionnaire utilized a five-point Likert scale (1 = Strongly Disagree, 2 = Disagree, 3 = Neutral, 4 = Agree, 5 = Strongly Agree). Additionally, an open-ended questionnaire was distributed to gather expert suggestions for further optimizing the intervention content.

### 2.3 Experimental intervention

#### 2.3.1 Pilates reformer training program

The 12-week Pilates Reformer training program in this study was based on STOTT Pilates principles, incorporating the specific characteristics of STOTT training movements. It adopted a neuromuscular functional training approach, focusing on enhancing technical performance through precise training methods (STOTT Pilates, 2024). This program was further developed through a combination of literature review (de Almeida et al., 2024; Parveen et al., 2023; Meikis et al., 2021), expert interviews, and professional recommendations from Pilates instructors. The Pilates Reformer training program included a systematic exercise regimen aimed at comprehensively improving participants’ physical fitness and performance capabilities.

The Experimental Group (EG) Pilates Reformer training program was divided into three phases: Phase 1: “Unconscious Incompetence” (1–4 weeks), Phase 2: “Conscious Incompetence” (5–8 weeks), Phase 3: “Conscious Competence” (9–12 weeks). The program consisted of three 90-min training sessions per week over a 12-week period. The exercise intensity and rest intervals were adjusted based on the difficulty of the movements and the resistance provided by the Reformer springs. The spring resistance and the unstable design of the sliding carriage on the Reformer offered both external resistance and support, enabling participants to perform exercises while overcoming instability. This design allowed them to focus more effectively on specific body parts or target muscle groups. Each phase was structured into three components: warm-up, Reformer-based movement training, and stretching. The exercises incorporated six different body positions: supine, prone, side-lying, kneeling, sitting, and standing. The content was progressively structured based on target muscles, joint movements, and increasing movement complexity. Throughout the functional training program, exercises were designed to promote continuous improvement, reflecting advancements in performance across all phases. A detailed description of the Pilates Reformer training program is provided in Table 2.

**TABLE 2:** Detailed description of the pilates reformer training program.

Training phase	Training content	Exercise name	Repetitions & sets	Rest interval
Phase 1 (1–4 weeks)	Warm-up	1. Supine rib cage breathing	10 reps *2 sets	10 s
		2. Pelvic placement principles		
		3. Rib cage placement principles		
		4. Scapular movement & stability principles		
		5. Head & cervical placement principles		
	Reformer exercises	1. Hip rolls	10 reps per side × 2 sets	20 s
		2. Lift & lower/parallel		
		3. Lift & lower/laterally rotated		
		4. Foot work/heels on bar		
		5. Foot work/high half hoe		
		6. Foot work/lower & lift		
		7. Arms pulling straps, long box-plow		
	Stretching exercises	1. Mermaid	10 reps × 2 sets	10 s
		2. Single thigh stretch		
Phase 2 (5–8 weeks)	Warm-up	1. Supine rib cage breathing	10 reps × 2 sets	10 s
		2. Pelvic placement principles		
		3. Rib cage placement principles		
		4. Scapular movement & stability principles		
		5. Head & cervical placement principles		
	Reformer exercises	1. Knee lift	10 reps × 2 sets	10 s
		2. Knee stretches/rounded back	5 reps × 2 sets	
		3. Knee stretches/flat back		
		4. Knee stretches/hover		
		5. Feet pulling/hamstring curls	10 reps × 2 sets	10 s
		6. Stomach massage/round back	10 reps × 2 sets	20 s
		7. Stomach massage/straight back		
		8. Stomach massage/reaching		
		9. Arms pulling straps, long box-airplane		
	Stretching exercises	1. Mermaid	10 reps × 2 sets	10 s
		2. Single thigh stretch		
Phase 3 (9–12 weeks)	Warm-up	1. Supine rib cage breathing	10 reps × 2 sets	10 s
		2. Pelvic placement principles		
		3. Rib cage placement principles		
		4. Scapular movement & stability principles		
		5. Head & cervical placement principles		
	Reformer exercises	1. Front splits	10 reps per side × 3 sets	10 s
		2. Back splits		
		3. Running	20 reps × 3 sets	10 s
		4. Lunge and extension	20 reps per side × 2 sets	10 s
		5. Arms pulling straps, long box-plow	10 reps × 3 sets	10 s
		6. Arms pulling straps, long box-airplane	10 reps × 3 sets	10 s
	Stretching exercises	1. Mermaid	10 reps × 2 sets	10 s
		2. Single thigh stretch		

#### 2.3.2 Training program for the control group

The control group’s regular training program began with a 10-min warm-up, followed by standardized practice of Latin dance basic steps and choreographed routines. The session concluded with a 10-min cool-down. Participants were instructed to train at an intensity of 60%–90% of their maximum effort, with the coach supervising and ensuring consistent attendance throughout the 12-week program. The control group (CG) training protocol primarily targeted single-axis local muscle groups through single-joint exercises. The main training consisted of 3–6 sets per exercise, with 10–20 repetitions per set, interspersed with rest intervals. During the 12-week program, the main training phase lasted 60 min per session (see Table 3 for details).

**TABLE 3:** Standard Training for control group.

Training content		Exercise name	Repetitions & sets	Rest interval
Warm-up		Step-touch, walking, mambo step, side step	2 reps × 2 sets	30 s
		Cross step, jumping jacks, back kick jumps		
Main training	Basic steps	Ronde chasse, ronde chasse + hip twist	10 reps × 6 sets	30 s
		Chasse, circular chase step with hip twist		
	Choreographed routine	1. Checked forward to foot change	1 round × 5 sets	30 s
		2. Ronde chasse		
		3. Side spot steps		
		4. Zigzag		
		5. Ronde chasse		
		6. Step taps (light tap steps)		
		7. Cuban breaks to step taps		
		8. Cuban breaks to step taps		
		9. Shakes		
		10. Kick forward		
		11. Turn to cross		
		12. Kick foot change		
		13. Cuban breaks		
		14. Kick foot change		
	Physical training	High knees, jumping jacks, frog jumps, V-ups, plank	20 reps × 3 sets	30 s
Stretching exercises		Relaxation of head, shoulders, waist, calves, wrists, ankles	1 round × 2 sets	30 s

During the entire intervention period, all participants were instructed not to engage in any form of structured training or high-intensity physical activity. They were also asked to avoid unhealthy lifestyle behaviors, such as binge eating, alcohol consumption, and smoking. Additionally, participants were advised to maintain consistent dietary and sleep patterns throughout the training period to minimize the potential influence of external lifestyle factors on the experimental outcomes.

### 2.4 Measurement procedure

#### 2.4.1 Experimental set-up

**FIGURE 1:**
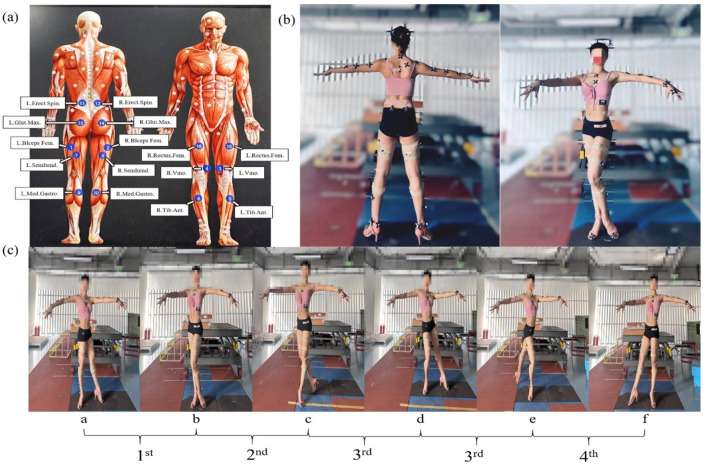
**(a)** Marked locations of selected muscles. **(b)** Joint marker placement. **(c)** Four phases and key moments of the Cha-Cha circular chase step technique.

A motion capture system comprising eight high-speed infrared cameras (OptiTrack, United States) with a sampling frequency of 200 Hz was used to record participants’ motion data during the execution of Cha-Cha circular chase steps. Consistent with previous studies, each participant was fitted with 39 spherical reflective markers (12.5 mm in diameter) during each trial to identify their movement patterns. This study adhered to the Surface Electromyography for the Non-Invasive Assessment of Muscles (SENIAM) guidelines for electrode placement on specific muscles and muscle regions (http://seniam.org/sensor_location.htm). Sixteen EMG sensors (Delsys, Boston, MA, United States) were attached to the muscle bellies of bilateral biceps femoris (BF), vastus medialis (VM), tibialis anterior (TA), semitendinosus (ST), gastrocnemius (GN), erector spinae (ES), gluteus maximus (GM), and rectus femoris (RF) to measure muscle activation. Reflective markers were placed at specific anatomical landmarks (see Figure 1a), and EMG sensors were positioned on the designated anatomical sites of target muscles (see Figure 1b).

#### 2.4.2 Data collection

##### 2.4.2.1 Biomechanical data acquisition

Data collection for this study was conducted in the Sports Biomechanics Laboratory at the Institute of Sports Artificial Intelligence, Capital University of Physical Education and Sports. Preparations before testing included the following steps: (1) Participant Information and Consent: Collected participant information, obtained signed informed consent forms, assessed health conditions, and provided a detailed explanation of the experimental procedures to the participants. (2) Camera Calibration and Coordinate System Definition: Calibrated the spatial positions of the cameras and defined the experimental coordinate system. (3) EMG Sensor Placement: Prepared the skin by removing excessive hair and cleaning the muscle belly areas with 75% medical alcohol to reduce resistance. Once the alcohol dried, electrodes were applied along the muscle fiber orientation and secured with kinesiology tape to ensure stability without affecting movement. After placement, EMG signals were tested to confirm the accuracy of the signal display during active muscle contraction. (4) Standardized Attire: Participants wore black or dark-colored tight-fitting clothing and shorts, along with professional Latin dance shoes, to ensure precise motion capture. (5) Marker Placement: A total of 39 reflective markers were attached to specific anatomical landmarks on the participants’ bodies according to the marker system. (6) Static Calibration: Participants maintained a T-pose (arms outstretched) under the motion capture system for static calibration. The corresponding data set was renamed and used for model scaling in OpenSim software. (7) Warm-Up and Movement Practice: Participants completed a 10-min warm-up session and rehearsed the experimental movements.

The test movement selected for this study was the Cha-Cha circular chase step, a routine commonly used in Latin dance competitions. The movement was divided into four phases and six key moments (see Figure 1c):

(1) Preparation Phase: Begins when the angle between the left knee external rotation and left ankle plantarflexion reaches its maximum (a) and ends when the angle of left knee flexion and adduction, along with the angles between the left hip and right ankle, reaches its minimum (b).
(2) Initiation Phase: Starts from the minimum angle of left knee flexion and adduction, as well as the angles between the left hip and left ankle (b), and ends when the left knee rapidly extends forward, the right knee flexes to touch the left knee pit, the foot contacts the ground, and the knee and ankle joint angles of both legs stabilize (c).
(3) Weight Transfer Phase: Begins when the knee and ankle joint angles of both legs stabilize (c) and ends when the right ankle dorsiflexion rapidly causes the heel to touch the ground, the right knee flexes, and the left ankle plantarflexes to complete the circular motion. This concludes when the left hip abduction and left knee external rotation angles reach their maximum (e).
(4) Completion Phase: Starts when the left ankle plantarflexion completes the circular motion and the left hip abduction and left knee external rotation angles reach their maximum (e) and ends when the right knee external rotation and right ankle plantarflexion angles reach their maximum (f).

##### 2.4.2.2 Test metrics

(1) Kinematic Metrics: Joint angles (°) and angular velocities (rad/s) of the hip, knee, and ankle in both the left and right legs. (2) Electromyographic (EMG) Metrics: Root Mean Square (RMS) amplitudes of muscle activation for the following muscles in both legs: Biceps Femoris (BF), Vastus Medialis (VM), Tibialis Anterior (TA), Semitendinosus (ST), Gastrocnemius (GN), Erector Spinae (ES), Gluteus Maximus (GM), Rectus Femoris (RF).

##### 2.4.2.3 Technical quality (TQ) data collection based on the WDSF 3.0 scoring system

To more effectively evaluate the impact of Pilates reformer training on the technical execution of the Cha-Cha circular chasing step, this study adopted the Technical Quality (TQ) evaluation criteria of the WDSF 3.0 scoring system. This system was employed to provide a standardized, scientific assessment of performance improvements following the intervention. To minimize subjective evaluation bias, a blinded assessment protocol was implemented during the scoring phase. Specifically, all performances were independently scored by third-party experts who were blinded to the participants’ group assignments and training interventions, thereby enhancing the objectivity and scientific rigor of the outcome evaluation.

**FIGURE 2:**
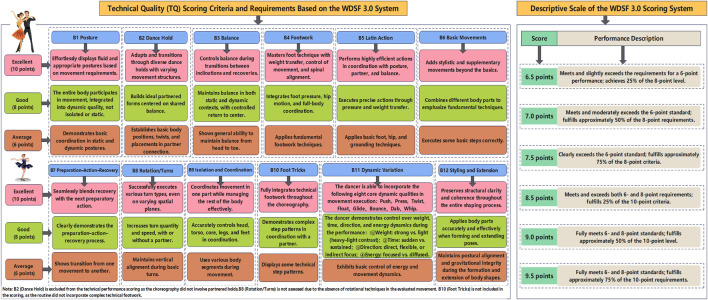
WDSF3.0 Latin dance technical quality scoring content, requirements and score description.

The WDSF 3.0 system refines and extends the structure of the earlier 2.1 version, offering a hierarchical scoring framework with both primary and secondary indicators that comprehensively reflect movement quality. It demonstrates an improved level of accuracy by increasing the granularity of score increments—from 0.5-point steps to 0.25-point steps—thus reducing the influence of evaluator subjectivity and strengthening the fairness and precision of the scoring process. The scoring rubric includes three qualitative levels of performance assessment: 6 points for average performance, 8 points for good performance, and 10 points for excellent performance. The detailed scoring criteria for each TQ sub-indicator are outlined in Figure 2.

#### 2.4.3 Data processing

(1) Filtering Valid Data: The raw experimental data may include invalid segments that must be removed, retaining only valid data. The starting point is defined as the first frame where the knee joint trajectory shows displacement, and the endpoint is the final frame where the participant completes the movement and returns to a stable upright position. Data before the starting point and after the endpoint are excluded.
(2) Repairing Missing Marker Data: Unnamed markers are assigned appropriate names, and naming errors are corrected. Additionally, during movement, some markers may not be captured by the cameras, leading to interruptions in trajectory data. These gaps are repaired using the Motive software.
(3) Filtering: After repairing the marker trajectories, Butterworth low-pass filtering is applied using Motive software to smooth the motion trajectory data. The cutoff frequency is set at 6 Hz.
(4) Data Export: Motion trajectory data are exported from Motive in C3D format. The C3D.Export.m script, provided by OpenSim, is run in Matlab R2021a to convert the C3D files into OpenSim-recognizable .trc and .mot files. The .trc file is used for model scaling and inverse kinematics, while the .mot file is used for inverse dynamics analysis.
(5) EMG Signal Preprocessing and Feature Extraction: Surface EMG signals are first processed with a fourth-order Butterworth band-pass filter, with a frequency range of 10–400 Hz. The signals are then full-wave rectified, followed by low-pass filtering with a cutoff frequency of 6 Hz. Finally, the signal amplitudes are normalized to the maximum root mean square (RMS) amplitude and further normalized using Maximum Voluntary Contraction (MVC) to determine muscle activation levels (Liang et al., 2024).

### 2.5 Statistical analysis

Data analysis and visualization were performed using Graph Prism 10.0 software (GraphPad Software, California, United States). All data were expressed as mean ± standard deviation (SD). Statistical analyses were conducted using SPSS software (Version 22 for Windows, Armonk, NY, United States), with the significance level set at p < 0.05. The normality of the data was assessed using the Shapiro-Wilk test. Paired-sample *t*-tests were used to compare pre- and post-training data within the experimental group and the control group. For between-group comparisons of changes (post-test–pre-test), independent-sample t-tests with two-tailed significance were employed.

## 3 Results

### 3.1 Results of the kinematics

**FIGURE 3:**
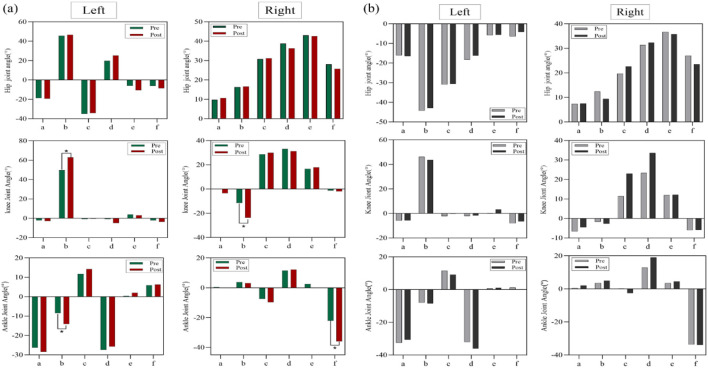
**(a)** Joint angle variations (°) of the hip, knee, and ankle during X-axis movements in the experimental group, **(b)** joint angle variations (°) of the hip, knee, and ankle during X-axis movements in the control group.

Figure 3 illustrates the kinematic differences between the experimental group and the control group at six key moments during the execution of Cha-Cha circular chase steps. Figure 3a: Shows the angle variations of the hip, knee, and ankle joints of both legs in the experimental group during X-axis movements at six key moments. The left knee joint displayed a significant difference at the moment of left knee flexion and adduction (P < 0.05), as did the left ankle joint at the same moment (P < 0.05). The left hip joint showed no significant differences at any key moment (P > 0.05), with mean values indicating minimal changes across key moments. The right knee joint showed a significant difference at the moment of left knee flexion and adduction (P < 0.05), and the right ankle joint exhibited a highly significant difference at the moment of right hip external rotation with heel lift-off (P < 0.05). The right hip joint also showed no significant differences at any key moment (P > 0.05), with mean values reflecting minimal changes across the key moments. Figure 3b: Displays the angle variations of the hip, knee, and ankle joints of both legs in the control group during X-axis movements. No significant differences were observed at any key moment for the hip, knee, or ankle joints of either leg (P > 0.05).

**FIGURE 4:**
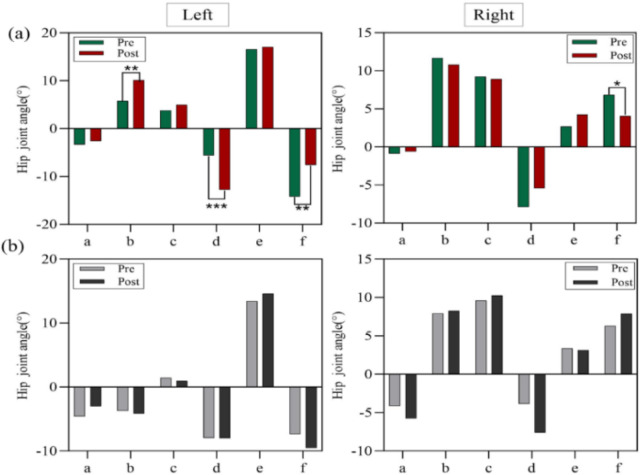
**(a)** Hip joint angle variations (°) in the experimental group during Y-axis movements, **(b)** hip joint angle variations (°) in the control group during Y-axis move.

Figure 4 illustrates the hip joint angle variations of the left and right legs at six key moments during Y-axis movements in the experimental and control groups. Figure 4a: Shows the angle variations in the experimental group. Significant differences were observed in the left hip joint at the moments of left knee flexion and adduction, left hip abduction with toe touch, and right hip external rotation with heel lift-off (P < 0.01). Although other key moments did not reach statistical significance, the data indicate notable changes at these moments. The right hip joint showed a significant difference at the moment of right hip external rotation with heel lift-off (P < 0.05). Figure 4b: Displays the angle variations in the control group. No significant differences were observed in the hip joint angles of the left or right leg at any key moment during Y-axis movements.

**FIGURE 5:**
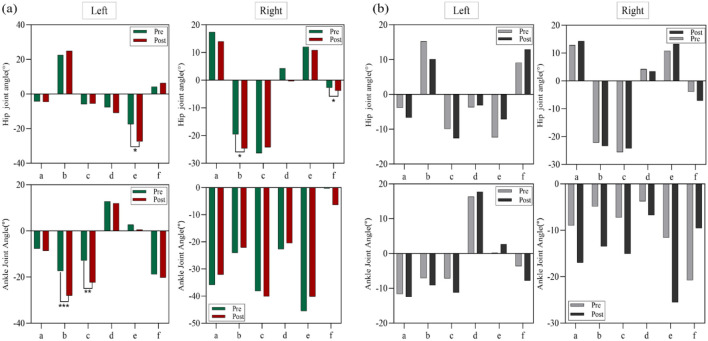
**(a)** Hip and ankle joint angle variations (°) in the experimental group during Z-axis movements, **(b)** hip and ankle joint angle variations (°) in the control group during Z-axis movements.

Figure 5 illustrates the angle variations of the hip and ankle joints in the left and right legs at six key moments during Z-axis movements for both the experimental and control groups. Figure 5a: Shows the angle variations in the experimental group. The left hip joint exhibited a significant difference at the moment when the left hip stopped the circular motion (P < 0.05). The left ankle joint showed highly significant differences at the moments of left knee flexion and adduction, and left knee extension (P < 0.01). The right hip joint displayed significant differences at the moments of left knee flexion and adduction, and right hip external rotation with heel lift-off (P < 0.05). Figure 5b: Displays the angle variations in the control group. No significant differences were observed at any key moment for the hip and ankle joints of the left or right leg during Z-axis movements (P > 0.05).

**FIGURE 6:**
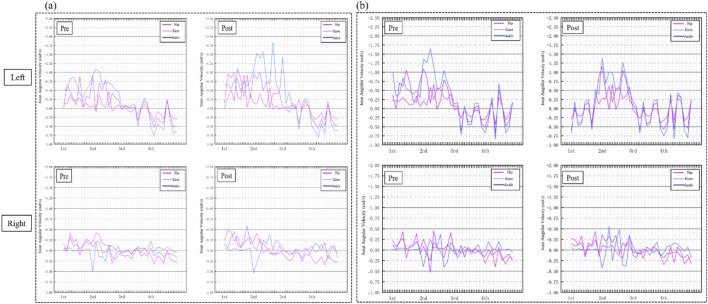
**(a)** Joint velocity curves of the hip, knee, and ankle in the left and right legs of the experimental group before and after training. **(b)** Joint velocity curves of the hip, knee, and ankle in the left and right legs of the control group before and after training.

Figure 6 illustrates the changes in angular velocity of the hip, knee, and ankle joints in the left and right legs during the four phases for the experimental and control groups. Figure 6a: Shows the experimental group’s joint velocity improvements following Pilates Reformer training. In the Preparation Phase, significant differences were observed in the left hip flexion velocity (P < 0.05), left knee flexion velocity (P < 0.05), and right hip flexion velocity (P < 0.05). In the Initiation Phase, significant differences were found in the left knee extension velocity (P < 0.05), left ankle flexion velocity (P < 0.05), and right knee flexion velocity (P < 0.05). However, no significant differences were observed in the hip flexion velocity of either leg (P > 0.05). In the Weight Transfer Phase, the left hip extension velocity showed a significant difference (P < 0.05), while no significant differences were observed in the velocities of the right hip, knee, or ankle joints. In the Completion Phase, significant differences were observed in the knee extension velocity of both legs (P < 0.05), but no significant differences were noted in hip flexion/extension or ankle extension velocities (P > 0.05). Figure 6b: Shows the control group’s joint velocities. Across all four phases—Preparation, Initiation, Weight Transfer, and Completion—no significant differences were observed in the angular velocities of the hip, knee, or ankle joints in either leg (P > 0.05).

### 3.2 Results of the electromyogram

**FIGURE 7:**
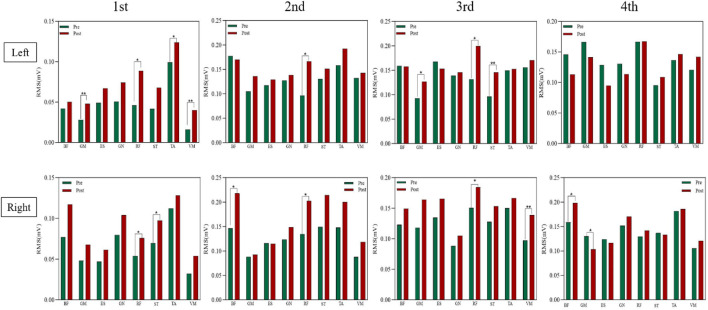
RMS values of lower limb muscles in the experimental group before and after training.

**FIGURE 8:**
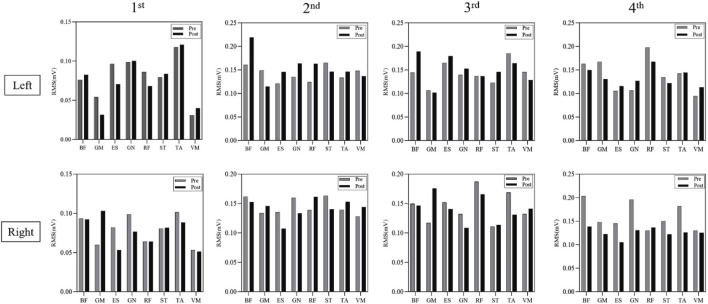
RMS values (mV) of lower limb muscles in the control group before and after training.

Figure 7 illustrates the significant increases in RMS normalized values of lower limb muscles in the experimental group following Pilates Reformer training. Preparation Phase: Paired-sample *t*-test results revealed significant post-training increases in the experimental group for the left leg’s gluteus maximus (P < 0.01), rectus femoris (P < 0.05), tibialis anterior (P < 0.05), and vastus medialis (P < 0.01) as well as the right leg’s rectus femoris (P < 0.05) and semitendinosus (P < 0.05). In contrast, no significant differences were observed in the control group before and after training. Initiation Phase: Significant increases were observed in the experimental group for the left leg’s rectus femoris (P < 0.05) and the right leg’s biceps femoris (P < 0.05) and rectus femoris (P < 0.05). Weight Transfer Phase: Post-training RMS values significantly increased in the experimental group for the left leg’s gluteus maximus (P < 0.05), rectus femoris (P < 0.05), and semitendinosus (P < 0.01) as well as the right leg’s rectus femoris (P < 0.05) and vastus medialis (P < 0.01). The control group exhibited no significant differences before and after training. Completion Phase: No significant changes were found in the left leg’s muscles of the experimental group. However, the right leg’s biceps femoris (P < 0.05) and gluteus maximus (P < 0.05) showed significant increases in RMS values. The control group displayed slight increases in integrated EMG values across all phases, but these did not reach statistical significance (Figure 8).

### 3.3 Comparative analysis of technical quality scores based on the WDSF 3.0 evaluation criteria

As shown in Table 4, according to the WDSF 3.0 technical quality (TQ) scoring criteria, the control group exhibited significant improvements in only two indicators during the performance of the Cha-Cha circular chasing step: B6 (basic movements, P < 0.01) and B7 (preparation–action–recovery, P < 0.05). In contrast, the experimental group demonstrated significant improvements across a broader range of indicators following the intervention, including B1 (posture, P < 0.05), B4 (foot movements, P < 0.05), B5 (Latin characteristics, P < 0.05), B6 (basic movements, P < 0.01), and B7 (preparation–action–recovery, P < 0.01). Additionally, the experimental group showed consistent improvements in average TQ scores across all evaluated components. These results suggest that a 12-week Pilates reformer training program can effectively enhance the technical execution of the Cha-Cha circular chasing step. Specifically, the training improved overall body coordination and postural control, allowing dancers to maintain proper alignment and demonstrate refined posture. It also enhanced the clarity and precision of footwork by integrating foot pressure distribution with coordinated joint (hip, knee, ankle) and body movements. Furthermore, the training facilitated efficient weight transfer and improved synergy between body mass and foot-ground interaction, thereby enhancing the execution of Latin dance elements. Finally, it promoted the coordination of different body segments, improved the articulation of foundational movements, and strengthened the clarity and continuity of movement transitions.

**TABLE 4:** Pre- and post-training technical quality (TQ) scores in the experimental and control groups.

Group	Indicator	Pre-training	Post-training	*t*-value	*P*-value
Experimental Group	B1 Posture	6.518 ± 0.223	7.558 ± 0.706	−2.177	0.048*
	B3 Balance	6.533 ± 0.160	6.733 ± 0.263	−1.036	0.359
	B4 Footwork	6.581 ± 0.208	7.473 ± 0.486	−3.223	0.032*
	B5 Latin characteristics	6.399 ± 0.122	7.171 ± 0.420	−3.392	0.027*
	B6 Basic movements	6.491 ± 0.177	7.223 ± 0.407	−2.326	0.040**
	B7 Preparation–action–recovery	6.508 ± 0.162	7.034 ± 0.379	−6.702	0.003**
	B9 Isolated–coordinated movements	6.501 ± 0.128	7.142 ± 0.853	−1.668	0.085
	B11 Dynamic variability	6.450 ± 0.150	6.826 ± 0.235	−1.911	0.064
	B12 Styling and extended styling	6.442 ± 0.083	7.202 ± 0.745	−1.854	0.069
Control group	B1 Posture	6.368 ± 0.144	6.548 ± 0.103	−1.668	0.085
	B3 Balance	6.528 ± 0.067	6.594 ± 0.110	−1.615	0.090
	B4 Footwork	6.526 ± 0.048	6.581 ± 0.139	−0.49	0.324
	B5 Latin characteristics	6.438 ± 0.117	6.569 ± 0.129	−1.646	0.087
	B6 Basic movements	6.468 ± 0.1187	6.618 ± 0.117	−5.857	0.004**
	B7 Preparation–action–recovery	6.518 ± 0.063	6.662 ± 0.094	−2.819	0.047*
	B9 Isolated–coordinated movements	6.446 ± 0.107	6.540 ± 0.114	−1.084	0.169
	B11 Dynamic variability	6.445 ± 0.108	6.566 ± 0.129	−1.389	0.118
	B12 Styling and extended styling	6.489 ± 0.093	6.606 ± 0.249	−0.931	0.202

## 4 Discussion

The primary aim of this study was to compare the effects of a 12-week Pilates Reformer training program and conventional technical training on the Cha-Cha circular chase step performance of Latin dance students. Following Pilates Reformer training, dancers demonstrated significant improvements in joint angles during the execution of the movement, including knee and ankle flexion-extension, hip abduction-adduction, and hip and ankle internal-external rotation. Additionally, angular velocities of the hip, knee, and ankle joints increased significantly, enhancing the coordination between muscle activation and joint responsiveness during the execution of technical movements. These improvements further highlighted the characteristic power and speed of the Cha-Cha. Moreover, Pilates Reformer training resulted in significantly greater muscle strength in the gluteus maximus, rectus femoris, tibialis anterior, vastus medialis, and semitendinosus compared to the conventional technical training group. The findings suggest that Pilates Reformer training may have a significant advantage over conventional technical training in improving the quality of Cha-Cha circular chase step performance. This approach holds promise as an effective training method for enhancing movement execution in Latin dance.

This study found significant improvements in the angles and velocities of the hip, knee, and ankle joints following training. Latin dance often involves extensive trunk rotations and hip twists, and the enhancement of joint angles and velocities contributes to improved performance and the ability to shift the center of gravity rapidly (Liu et al., 2022). Notably, in lower limb joints, studies have shown a strong correlation (cross-correlations ≥0.63) between flexion-extension relationships across joints during Cha-Cha movements at high, medium, and low skill levels. Additionally, research has demonstrated that increases in movement amplitude and speed are closely linked to improvements in dance quality and aesthetics (Neave et al., 2010; Torrents et al., 2013). Visually, larger internal and external rotation angles of the lower limb joints enhance the three-dimensional appearance of movements and emphasize the elongation of leg lines. Firstly, Muscle Activation and Force Generation. Pilates Reformer training strengthens the inner thigh muscles, enabling the left leg to adduct effectively toward the body’s neutral position during rapid hip and knee flexion. Subsequently, the knee’s forward motion drives a seamless extension of the lower leg, while the ankle’s increased flexion speed efficiently absorbs and transmits force, allowing the foot to stabilize firmly against the ground, creating effective ground pressure. Secondly, Enhanced Joint Mobility. Compared to conventional training, Pilates Reformer training significantly improves knee and ankle flexion-extension, hip abduction-adduction, and hip and ankle internal-external rotation during the Cha-Cha circular chase step. These enhancements contribute to the improved execution quality of technical movements. Finally, Muscle-Specific Activation. Pilates Reformer training effectively activates the various muscles required for executing the Cha-Cha circular chase step (Powers et al., 2007). By improving joint responsiveness, the training amplifies the distinctive power and speed characteristics of the Cha-Cha.

Previous studies have demonstrated that an 8-week Reformer training program significantly improved muscle strength in adolescent baseball players (Park et al., 2020). Similarly, this study found that Pilates Reformer training significantly enhanced lower limb strength in both the left and right legs of Latin dancers. Furthermore, elite-level Latin dancers exhibit significantly greater muscle strength compared to advanced (national-level) performers, even though both groups are professional dancers (Wanke et al., 2018). This suggests that Pilates training may play a positive role in enhancing muscle strength among Latin dancers. Additionally, muscle strength has been shown to be strongly associated with the performance of Latin dancers, particularly in key movements such as turning, rapid directional changes, and balance adjustments (Zaletel, 2020). In summary, improving muscle strength is likely one of the critical factors in enhancing the technical performance of Latin dancers (Ödemiş et al., 2022).

This study has several limitations that should be addressed in future research. Firstly, the sample size was relatively small, and all participants were recruited from a single research center, which limits the representativeness and generalizability of the findings. Secondly, although this is the first study to investigate the impact of Pilates reformer training on the biomechanics of the Latin Cha-Cha circular chasing step, potential confounding variables were not sufficiently controlled during the intervention. Specifically, participants’ dietary habits, sleep quality, daily stress levels, other physical activities, and caloric intake and expenditure were not recorded or monitored, which may have influenced the training outcomes. Thirdly, while outcome assessments were conducted by independent experts blinded to group allocation to reduce subjective bias, both participants and intervention providers were aware of the group assignments, potentially introducing observer effects. Therefore, future studies should aim to recruit a larger and more diverse sample, including Latin dancers of different genders and training levels. In addition, daily behavioral records and energy balance monitoring mechanisms should be incorporated to enhance the scientific rigor, explanatory power, and applicability of the findings.

## 5 Conclusion

This study demonstrates that compared to conventional technical training, integrating Pilates Reformer training into foundational Latin dance programs has a more profound and significant impact on dancers. Pilates Reformer training not only substantially improved joint flexibility—such as knee adduction and ankle dorsiflexion during X-axis movements, hip adduction, abduction, and external rotation during Y-axis movements, and hip internal and external rotation along with ankle dorsiflexion during Z-axis movements—but also enhanced the quality of technical movements, expanded movement range, and showcased the aesthetic beauty of dance postures. Additionally, Pilates Reformer training significantly activated key lower limb muscles, including the gluteus maximus, rectus femoris, tibialis anterior, and vastus medialis, contributing to safer training practices. Therefore, incorporating Pilates Reformer training into regular Latin dance curricula can effectively improve students’ technical performance while significantly reducing injury risk. This training method is also highly beneficial for enhancing muscle strength, body control, joint flexibility, movement range, and breathing patterns in Latin dance movements.

## Data Availability

The original contributions presented in the study are included in the article/supplementary material, further inquiries can be directed to the corresponding author.
